# Democratizing cloud data lake analytics: natural language access to Apache Iceberg via LLM agents

**DOI:** 10.3389/fdata.2026.1785710

**Published:** 2026-06-08

**Authors:** Vipin Kataria, Nitin Kumar

**Affiliations:** 1 Picarro Inc., Santa Clara, CA, United States; 2 Marriott International, Bethesda, MD, United States

**Keywords:** Apache Iceberg, conversational data access, data lakes, LangChain, large language models, natural language processing, semantic layer, SQL-free querying

## Abstract

Business analysts and non-technical users need insights from enterprise data lakes but lack SQL expertise to query them directly. While large language models (LLMs) can translate natural language to SQL, existing text-to-SQL approaches face critical limitations: severe SQL injection vulnerabilities, inability to leverage data-lake-specific features like time-travel queries, and inconsistent metric definitions across organizations. We present the LangChain Iceberg Toolkit, enabling users to query Apache Iceberg data lakes through natural language conversations with LLM agents, no SQL knowledge required. Users ask questions in plain English (e.g., “What was revenue last quarter?”), and the system automatically: (1) interprets intent using LLMs, (2) selects appropriate tools from a YAML-based semantic layer mapping business terms to data structures, (3) executes queries through a hybrid architecture combining PyIceberg's type-safe API (for security) with DuckDB's SQL engine (for complex analytics), and (4) returns formatted answers with business context. Our evaluation demonstrates 100% success across 100 systematically designed queries leveraging semantic layer integration for consistent metric definitions. Critically, in direct comparison against a schema-aware text-to-SQL baseline on the same query set, our system achieves a 33 percentage-point accuracy improvement (100% vs. 67%) while reducing SQL injection attack success rate from the 99% reported in prior text-to-SQL research to 0% across both execution paths. End-to-end query latency averages 2.6 seconds on 15.1M records, with partition pruning eliminating 90%+ of scanned data files. The hybrid execution architecture prevents SQL injection vulnerabilities through type-safe query construction for simple queries and controlled, pre-validated SQL execution for complex analytics. Users receive data insights through conversational interfaces without writing SQL, understanding schemas, or knowing technical implementation details. We provide a production-ready, open-source implementation demonstrating practical viability for democratizing enterprise data access.

## 1 Introduction

### 1.1 The challenge: data lakes require technical expertise

Modern enterprises store vast amounts of data in lakehouse architectures built on open table formats like Apache Iceberg (Zaharia et al., 2021; Okolnychyi et al., 2024). These systems provide powerful capabilities, ACID transactions, time-travel queries, schema evolution, but accessing them remains challenging for non-technical users. Business analysts who need to answer questions like “What was revenue last quarter?” or “Which regions saw declining sales?” must either learn SQL or wait for data teams to write queries for them.

This challenge extends beyond individual organizations: Taufer et al. (2026) identify democratizing data access as a strategic priority for scientific and enterprise innovation, noting that technical barriers to data querying represent one of the most significant bottlenecks to knowledge discovery and decision-making at scale. The fundamental bottleneck is that the people with domain expertise (business users) cannot directly access the data they need to make decisions. Traditional SQL-based approaches require users to understand complex table schemas, master query syntax, and handle technical concepts like partitioning and joins. While SQL is powerful for data professionals, it remains a significant barrier for the majority of business users (Hong et al., 2024).

Beyond technical complexity, organizations face a semantic consistency problem. The same business concept (e.g., “revenue” or “active customer”) may be calculated differently by different teams, leading to inconsistent reporting and conflicting insights (Janssen et al., 2020). Without centralized, authoritative metric definitions, organizations struggle to establish a single source of truth.

### 1.2 The promise and pitfalls of LLM-based data access

Large language models (LLMs) like GPT-4 and Claude have demonstrated remarkable capabilities in understanding natural language and generating code (Brown et al., 2020; Touvron et al., 2023). These capabilities suggest a promising solution: users could ask data questions in plain English, and LLMs could translate them to SQL automatically. This “text-to-SQL” approach has attracted significant research attention, with benchmarks like Spider evaluating systems' ability to generate correct SQL from natural language (Yu et al., 2018; Lei et al., 2024).

However, despite recent advances, text-to-SQL approaches face critical limitations that prevent their deployment in production environments:

**Security vulnerabilities**: Dynamic SQL generation from user input is vulnerable to injection attacks. Recent research demonstrates 99% attack success rates against text-to-SQL systems, where malicious users can extract sensitive data or damage databases (Zhang et al., 2023). Traditional approaches that concatenate user input into SQL strings create severe security risks.

**Lack of business context**: LLMs generating SQL from natural language lack awareness of organizational metric definitions. When a user asks about “revenue,” the LLM must guess which tables to use, how to calculate the metric, and what filters to apply. Different teams may get different answers for the same question, undermining trust in the system.

**Limited format awareness**: General-purpose text-to-SQL systems cannot leverage data-lake-specific features like Iceberg's time-travel queries, snapshot isolation, or partition pruning. They treat Iceberg tables as generic SQL tables, missing optimization opportunities and unique capabilities.

**Semantic inconsistency**: Without a centralized semantic layer, LLMs must infer metric calculations from table schemas and column names. Prior research demonstrates significant accuracy improvements when semantic layers provide business context to LLMs (Schwanke, 2024), but existing approaches lack integration with modern lakehouse formats.

### 1.3 Our solution: natural language access through semantic tools

This paper presents the LangChain Iceberg Toolkit, a system that enables non-technical users to query Apache Iceberg data lakes through natural language conversations with LLM agents, without writing SQL or understanding technical data structures. The key insight is that users should interact with *business concepts* (metrics, dimensions, and time periods) rather than technical constructs (tables, columns, SQL syntax).

#### 1.3.1 How it works: a natural language query workflow

Consider a business analyst who needs to understand air quality trends but lacks SQL knowledge. With traditional SQL approaches, they would need to write:


SELECT city, AVG(o3_ppb) as avg_ozone
FROM epa.daily_summary
WHERE date >= '2024-11-01' AND date  <
'2024-12-01'
  AND city = 'Los Angeles'
  AND o3_ppb IS NOT NULL
GROUP BY city


This requires knowing: the table name (epa.air_quality_readings), the column for ozone (o3_ppb), proper date filtering syntax, NULL handling, and SQL aggregation syntax.

With our toolkit, the same analyst simply asks:

**User:** “*What was the average ozone level in Los Angeles last month?”*

The system automatically:

1. **LLM interprets the natural language query**: Identifies intent (get average ozone), location (Los Angeles), and time period (last month)
2. **Semantic layer maps business terms to technical structures**: “ozone level” → o3_ppb column, “average” → AVG aggregation, “last month” → appropriate date range
3. **LLM selects appropriate tool**: Finds get_avg_ozone_level() from auto-generated semantic tools
4. **Tool executes query**: Routes to PyIceberg API (type-safe, no SQL injection risk)
5. **User receives formatted answer**: “*Average ozone level in Los Angeles (November 2024): 48.3 ppb. EPA threshold: 70 ppb. Status: Within acceptable limits.”*

The user receives their answer without writing SQL, understanding table schemas, or knowing technical implementation details. The semantic layer ensures “ozone level” consistently maps to the correct column, applies proper filters, and formats results with relevant business context (EPA threshold comparison).

For more complex queries requiring multi-table JOINs, the system routes execution to DuckDB's SQL engine while maintaining security through controlled, validated execution, not dynamic SQL generation from user input.

#### 1.3.2 Key innovations

Our approach addresses the limitations of existing text-to-SQL systems through several key innovations:

**First: natural language interface with semantic understanding:** We provide a conversational interface where users ask questions in plain English. A YAML-based semantic layer maps business terminology to technical data structures, ensuring LLMs interpret queries correctly. Users interact with familiar business concepts (revenue, customer segment, time period) rather than technical constructs (table names, column names, SQL syntax).

**Second: automatic tool generation from business metrics:** Organizations define business metrics once in a declarative YAML format. The system automatically generates LangChain tools for each metric, eliminating manual tool development while ensuring consistent metric calculations enterprise-wide. When users ask about “revenue,” all teams get the same answer using the same calculation.

**Third: secure hybrid execution architecture:** Instead of generating arbitrary SQL from user input, we route queries through two secure execution paths: PyIceberg's type-safe Python API for simple queries (eliminating SQL injection entirely) and DuckDB's SQL engine for complex analytics (with controlled, validated execution). This provides both security (type-safe for simple queries) and analytical power (SQL for complex JOINs and aggregations).

**Fourth: natural language access to advanced features:** Users can ask time-travel questions like “How does today compare to last year?” without understanding Iceberg's snapshot architecture. The system handles technical details (snapshot IDs, timestamp resolution, and historical data retrieval) transparently while users interact in familiar business language.

**Fifth: production-ready LLM agent integration:** We demonstrate end-to-end natural language workflows through LangChain agents that automatically discover available tools, interpret user intent, select appropriate tools, extract parameters, and format results, all without requiring users to understand the underlying system architecture.

### 1.4 Contributions

The main contributions of this work are:

1. **Natural language data access for non-technical users:** We present a production-ready system enabling non-technical users to query Apache Iceberg data lakes using plain English, eliminating SQL barriers through automatic tool selection and semantic layer integration.
2. **Semantic layer for consistent business metrics:** We introduce a YAML-driven semantic layer that maps business terminology to technical data structures, ensuring all users get consistent answers for the same business questions. This prevents the “which revenue calculation?” problem common in organizations.
3. **Secure hybrid architecture enabling SQL-free access:** We eliminate SQL injection vulnerabilities while maintaining analytical power through a dual-execution approach: type-safe PyIceberg API for simple queries (no SQL generation), controlled DuckDB SQL for complex analytics (validated, not user-generated). Users benefit from both security and analytical capability without writing SQL.
4. **Automatic tool generation from metric definitions:** We show how semantic layer configurations automatically translate into executable LangChain tools with proper schemas and descriptions, enabling dynamic tool generation without code changes. Organizations define metrics once; the system generates conversational interfaces automatically.
5. **Natural language access to time-travel queries:** We demonstrate how Iceberg's snapshot-based architecture can be exposed through natural language interfaces, enabling users to ask “How does today compare to last year?” without understanding snapshot IDs or time-travel SQL syntax.
6. **Production framework with real-world evaluation:** We provide the first enterprise-grade toolkit enabling non-technical users to query Apache Iceberg data lakes through conversational interfaces, with comprehensive documentation, error handling, and real-world deployment examples (GitHub + PyPI).
7. **Empirical comparative evaluation against baselines:** We quantify the contribution of our architectural choices through direct comparison against schema-free text-to-SQL, schema-aware text-to-SQL, and expert manual SQL baselines on the same 100-query evaluation suite, establishing the empirical novelty of the proposed design.

### 1.5 Paper organization

The remainder of this paper is organized as follows: Section 2 provides background on Apache Iceberg, LLMs, and semantic layers. Section 3 describes our system architecture and how it enables natural language data access. Section 4 presents our evaluation including comparative analysis against baseline approaches and security validation. Section 5 discusses implications for democratizing data access in enterprises. Section 6 concludes and outlines future work.

## 2 Background and related work

### 2.1 The SQL barrier for non-technical users

While SQL remains the dominant language for data querying, it presents significant challenges for business users who must understand technical concepts (schemas, joins, and aggregations), precise syntax, and database-specific optimizations (Hong et al., 2024). This creates dependency on data teams for every question, limiting data democratization in enterprises.

### 2.2 Text-to-SQL: promise and limitations

The text-to-SQL problem, translating natural language questions into SQL queries, has attracted significant research attention (Yu et al., 2018; Gan et al., 2021; Dong et al., 2023). The Spider benchmark evaluates systems on their ability to generate correct SQL from natural language across diverse schemas (Lei et al., 2024). Recent approaches explore few-shot prompting with LLMs (Sun et al., 2023) and chain-of-thought reasoning, but comprehensive surveys reveal text-to-SQL remains far from solved (Li et al., 2024; Luo et al., 2025).

Commercial systems implementing text-to-SQL include Thoughtspot's natural language search (Thoughtspot, 2024) and Microsoft Copilot for SQL analytics (Microsoft, 2024). However, these systems face the limitations outlined above: SQL injection vulnerabilities when generating dynamic queries, lack of semantic consistency without centralized metric definitions, and inability to leverage lakehouse-specific features like Iceberg's time-travel capabilities.

Critical limitations prevent production deployment: (1) **Security**: Dynamic SQL generation from user input creates 99% attack success rates for SQL injection (Zhang et al., 2023). (2) **Semantic consistency**: Without business context, LLMs generate different SQL for the same business question, leading to inconsistent answers. (3) **Format awareness**: General text-to-SQL lacks awareness of data-lake-specific features like time-travel queries. (4) **Accuracy**: Complex schema relationships lead to incorrect queries (Gan et al., 2021).

### 2.3 LLM-based agents and tool use

Recent advances demonstrate LLMs' ability to use tools to accomplish tasks (Wang et al., 2024; Yao et al., 2023). Rather than generating arbitrary code, LLM agents can select from predefined tools, invoke them with appropriate parameters, and interpret results. The ToolLLM framework shows how LLMs can master thousands of real-world APIs (Qin et al., 2023), while research on multi-agent systems (Zhou et al., 2024) and tool learning (Xu et al., 2025) demonstrates practical applications.

This tool-based approach offers a solution to text-to-SQL limitations: instead of generating arbitrary SQL from natural language, LLMs can select from validated, pre-defined query tools. However, existing tool-use frameworks (ToolLLM, LangChain) provide generic tool orchestration without domain-specific integration for data lakes or semantic business metrics. Our work extends this approach by automatically generating data query tools from semantic layer definitions, creating a bridge between LLM agents and Iceberg data lakes.

### 2.4 Semantic layers for business context

Semantic layers map business terminology to technical data structures, providing a bridge between how users think (business concepts) and how data is stored (tables and columns) (Databricks, 2024; IBM, 2024). They define authoritative metric calculations, ensuring “revenue” means the same thing across the organization.

Existing semantic layer implementations include dbt Semantic Layer for dbt-based data transformations (dbt Labs, 2024), Looker's LookML tightly coupled to Looker (Google Cloud Looker, 2024), and Cube.dev requiring JavaScript/YAML configuration (Cube Dev, 2024). However, most existing semantic layers are tightly coupled to specific query engines or require extensive manual configuration (Databricks, 2024).

Research demonstrates that LLM accuracy in answering data questions can improve by up to 300% when integrating with a semantic layer instead of directly targeting database tables (Schwanke, 2024). The semantic layer provides essential business context that enables LLMs to interpret user intent correctly and prevents hallucinations by enforcing queries through predefined business definitions (AtScale, 2024).

Our approach provides a flexible, YAML-based semantic layer that automatically generates conversational query interfaces through tool generation, addressing the configuration complexity of existing solutions while enabling natural language access.

### 2.5 Apache Iceberg and lakehouse architecture

Apache Iceberg is an open table format designed for large analytic datasets in data lakes (Okolnychyi et al., 2024). Developed at Netflix and used to manage over 100 petabytes of data, Iceberg provides ACID transactions, time-travel queries, schema evolution, and hidden partitioning, features addressing critical limitations of traditional data lake formats (Jain et al., 2023).

Current approaches to querying Iceberg tables require SQL knowledge: Apache Spark SQL, Trino (Trino Project, 2024), Presto, and Amazon Athena all provide SQL interfaces, while PyIceberg offers a Python API for programmatic access. None provide conversational natural language interfaces with semantic layer integration for non-technical users.

Lakehouse architectures combine the low-cost storage of data lakes with ACID transactions and performance of data warehouses (Zaharia et al., 2021). Case studies demonstrate 50% performance improvements and 10x storage reduction when adopting lakehouse architectures (Begoli et al., 2021). Our work makes Iceberg's advanced features (time-travel, snapshot isolation, and partition pruning) accessible through natural language interfaces.

### 2.6 DuckDB: analytical database with Iceberg integration

DuckDB is an embeddable analytical database designed for OLAP workloads (Raasveldt and Mühleisen, 2019). DuckDB's Iceberg extension provides zero-copy data access through the iceberg_scan() function, reading directly from Iceberg's Parquet files while respecting partition pruning (Raasveldt and Mühleisen, 2020).

This integration addresses a key limitation: while PyIceberg's native API provides security (type-safe, no SQL injection), it lacks support for complex multi-table JOINs common in analytical workloads. DuckDB fills this gap while maintaining performance through zero-copy access, but still requires SQL knowledge. Our toolkit makes DuckDB's analytical capabilities accessible through natural language.

### 2.7 Gap in existing work

While text-to-SQL systems enable natural language database access and semantic layers provide business context, no existing work combines:

1. Natural language query interface (text-to-SQL capability).
2. Semantic layer integration (consistent business metrics).
3. Native Apache Iceberg support (time-travel, partition pruning).
4. Secure hybrid execution (type-safe API + controlled SQL).
5. Production-ready implementation (open-source toolkit).

Text-to-SQL systems like Thoughtspot and Microsoft Copilot lack semantic layers and Iceberg-specific features. Semantic layer products like dbt Semantic Layer and Looker lack natural language interfaces and require SQL knowledge. Iceberg access tools (Spark SQL, Trino, and PyIceberg) require technical expertise. Our toolkit is the first to integrate all these capabilities in a production-ready framework enabling truly SQL-free data access for non-technical users.

## 3 Methods: enabling natural language data access

### 3.1 System overview: from natural language to answers

**Figure 1:**
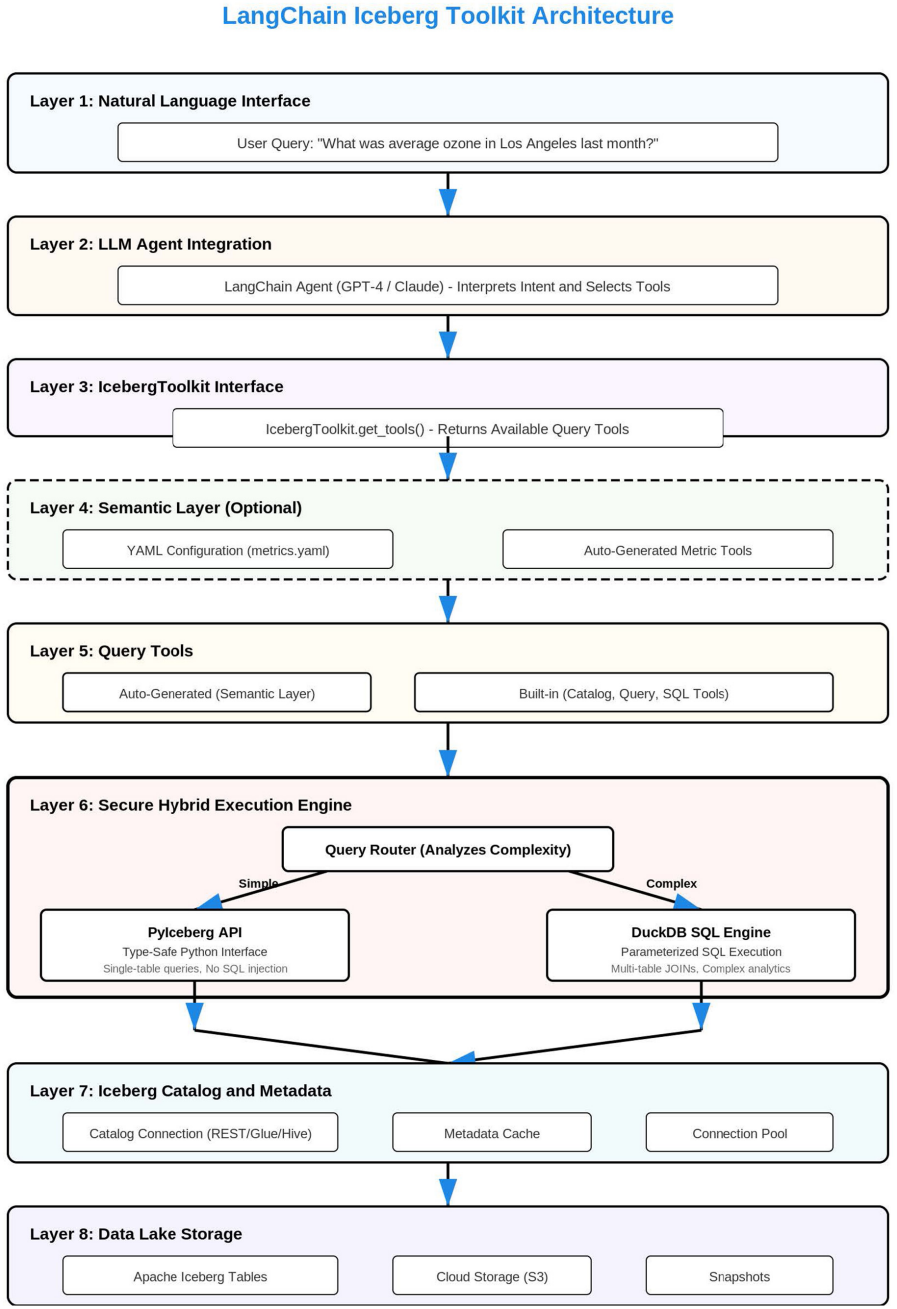
System architecture enabling natural language data access. Queries flow top-to-bottom through LLM agents (Layer 2), semantic layer (Layer 4, optional), and query tools (Layer 5) to the hybrid execution engine (Layer 6), which routes to PyIceberg for simple operations or DuckDB for complex analytics. The system accesses data through Iceberg's catalog layer (Layer 7) and cloud storage (Layer 8).

The LangChain Iceberg Toolkit enables users to ask data questions in plain English and receive formatted answers without writing SQL. Figure 1 illustrates the system architecture supporting this natural language workflow.

The architecture has eight layers supporting natural language workflows:

**Layer 1 - Natural language interface:** Users ask questions in plain English without SQL knowledge. Example: “What was average ozone in Los Angeles last month?”

**Layer 2 - LLM agent integration:** Conversational agents (GPT-4, Claude) interpret user intent via external API calls, discover available tools, and extract parameters from natural language queries.

**Layer 3 - IcebergToolkit interface:** The toolkit's get_tools() method provides the central integration point, returning all available query capabilities to LLM agents.

**Layer 4 - Semantic layer (optional):** Business metrics defined in YAML automatically generate query tools. Organizations define metrics once (e.g., “revenue,” “active customers”); the system generates conversational interfaces automatically. The semantic layer routes to either PyIceberg (expression-based metrics) or DuckDB (formula-based metrics with SQL).

**Layer 5 - Query tools**: Two categories serve different needs: (1) auto-generated tools from the semantic layer for business metrics, and (2) built-in exploratory tools for schema discovery, *ad-hoc* queries, and time-travel operations.

**Layer 6 - Secure hybrid execution engine:** The core innovation, queries route intelligently through two execution paths. The Query Router analyzes complexity and directs to: (1) PyIceberg API for simple operations (type-safe, eliminates SQL injection entirely), or (2) DuckDB SQL Engine for complex analytics requiring JOINs (parameterized queries prevent SQL injection while enabling analytical power). This hybrid approach provides both security and capability.

**Layer 7 - Iceberg catalog and metadata:** Catalog connections (REST, AWS Glue, Hive, and Nessie) manage table metadata access. Metadata caching reduces latency; connection pooling handles concurrent requests.

**Layer 8 - Data lake storage:** Apache Iceberg tables stored in cloud object storage (S3, HDFS, and Azure Blob Storage). Our toolkit accesses snapshot metadata at this layer to expose time-travel functionality through natural language tools, transparently to the user.

### 3.2 Natural language query workflow

**Figure 2:**
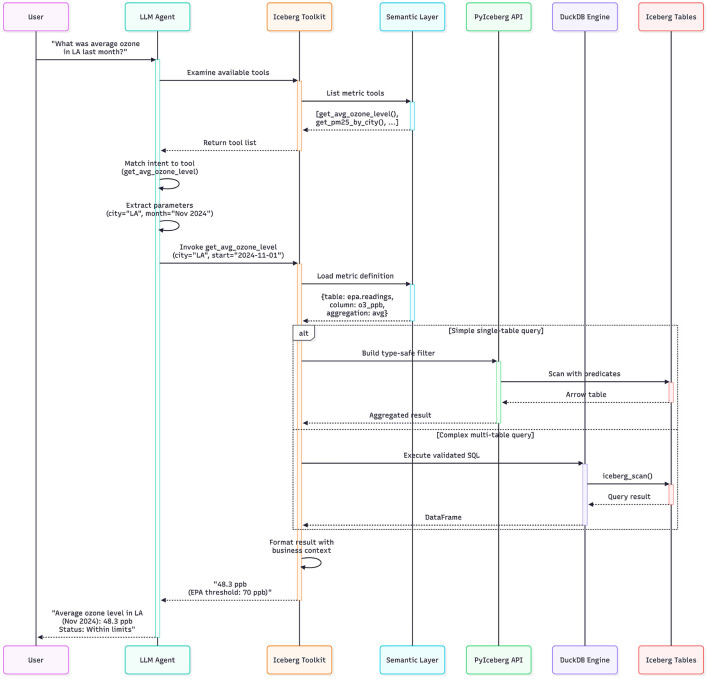
Complete natural language query execution flow. LLM agent interprets intent, semantic layer provides business context, hybrid execution ensures security and analytical power, and users receive formatted answers without SQL knowledge.

Figure 2 illustrates the complete query execution flow from natural language question to formatted answer.

The workflow proceeds through five distinct stages, as illustrated in Figure 2. The process begins when a non-technical user submits a plain English question through the conversational interface (Step 1). The LangChain agent receives this input and queries the semantic layer to identify available tools, matching the user's intent to the most appropriate metric definition (Step 2). Once matched, the semantic layer provides business context, column mappings, aggregation rules, and formatting instructions, which the toolkit uses to construct a type-safe query (Step 3). The hybrid execution engine then routes the query: simple single-table operations proceed through PyIceberg's Python API, eliminating SQL entirely, while multi-table analytics route through DuckDB's SQL engine using pre-validated queries sourced from the semantic layer (Step 4). Finally, results are formatted with business context and returned to the user in plain language (Step 5). At no point does the user write SQL, inspect a schema, or interact with the underlying execution engine directly.

#### 3.2.1 Step 1: user asks question in plain English

A business analyst with no SQL knowledge asks:

“*What was the average ozone level in Los Angeles last month?”*

No technical knowledge required, just a natural business question. With the question received, the system's first task is to map this unstructured natural language to a structured tool invocation. This is the responsibility of the LLM agent, described next.

#### 3.2.2 Step 2: LLM agent interprets intent

The LangChain agent:

1. Examines available tools from the semantic layer.
2. Identifies relevant metric: get_avg_ozone_level() from auto-generated tools.
3. Extracts parameters from natural language:
  - Location: “Los Angeles” → city parameter.
  - Time: “last month” → date range (November 1–30, 2024).
  - Metric: “average ozone level” → avg aggregation.
4. Constructs tool invocation: get_avg_ozone_level(city="Los Angeles", start_date="2024-11-01", end_date="2024-11-30").

Tool invocation alone is not sufficient, the agent has selected *what* to compute, but the semantic layer determines *how* to compute it consistently across users.

#### 3.2.3 Step 3: semantic layer provides business context

The YAML semantic layer defines the metric:


metrics:
  - name: avg_ozone_level
    description: "Average ozone concentration
    in parts per billion"
    table: epa.daily_summary
    aggregation: avg
    column: o3_ppb
    filters:
      - column: o3_ppb
        operator: "IS NOT NULL"
    format: decimal_2
    business_context: "EPA threshold: 70 ppb"
 

This ensures:

- “Ozone level” consistently maps to o3_ppb column.
- Proper NULL handling.
- Results formatted with 2 decimal places.
- Answer includes EPA threshold for context.

With the metric definition resolved, the toolkit must now execute the query against the underlying data, the point at which security considerations dominate the design.

#### 3.2.4 Step 4: secure query execution

The toolkit:

1. Routes to PyIceberg execution path (simple aggregation, no JOIN needed).
2. Constructs type-safe filter expressions (no SQL string generation):

from pyiceberg.expressions import And,
EqualTo, GreaterThanOrEqual
from pyiceberg.expressions import LessThan
filter_expr = And(
  EqualTo("city", "Los Angeles"),
  GreaterThanOrEqual("date", "2024-11-01"),
  LessThan("date", "2024-12-01"),
  NotNull("o3_ppb")
)
 
3. Executes query: table.scan(row_filter=filter _expr).to_arrow().
4. Calculates aggregation in Python.
5. Formats result with business context.

No SQL injection risk, all filtering through type-safe expressions. Raw query results, however, are insufficient for non-technical users; the final stage transforms them into business-contextualized answers.

#### 3.2.5 Step 5: user receives formatted answer

“*Average ozone level in Los Angeles (November 2024): 48.3 ppb*

*EPA threshold: 70 ppb*

*Status: Within acceptable limits”*

The user received their answer without:

- Writing SQL.
- Knowing the table name (epa.air_quality_readings).
- Knowing the column name (o3_ppb).
- Understanding date filtering syntax.
- Manually formatting results.
- Looking up EPA thresholds.

This five-stage flow, natural language input, intent resolution, semantic mapping, secure execution, and contextualized output, occurs on every query without user awareness. The user perceives only the input (their question) and output (the answer with business context). The intermediate stages are invisible by design: a user who already understood the snapshot architecture, schema layout, or routing decisions would have little need for the system in the first place.

#### 3.2.6 LLM-Iceberg integration mechanics

The integration between the LLM agent and PyIceberg is mediated by a tool registry generated at toolkit initialization rather than at query time. The MetricToolGenerator inspects each YAML metric definition once and produces a LangChain BaseTool instance whose name, description, and Pydantic input schema are derived deterministically from the metric configuration. Tool names follow the convention get_ <metric_name> (e.g., get_avg_ozone_level), and tool descriptions are constructed from the metric's description field combined with structured input documentation of accepted parameters such as date_range and group_by.

Critically, the tool registry encodes the execution path at registration time: metrics with expression definitions become PyIceberg tools, metrics with formula definitions or those flagged with requires_join become DuckDB tools, and the LLM agent sees a uniform tool interface across both paths. This early-binding decision is what allows the security boundary, type-safe PyIceberg expressions for simple queries and pre-validated DuckDB SQL templates for complex ones, to be enforced architecturally rather than relying on the LLM to comply with safety constraints at query time.

The toolkit additionally abstracts catalog implementation details: the same registered tools operate unchanged across REST, Hive, AWS Glue, Nessie, SQL, and in-memory catalog backends, since all catalog interactions are mediated by PyIceberg's catalog abstraction. The LLM never sees catalog configuration, schema layout details, or partition keys; its view of the data is exactly the semantic layer. This separation is what enables tool selection to remain stable across schema evolution: only the YAML semantic layer must be updated when columns change, not the LLM prompt or the agent's reasoning logic.

### 3.3 Semantic layer: mapping business terms to technical structures

The semantic layer is the key enabler of natural language access. It bridges the gap between how users think (business concepts) and how data is stored (technical structures).

#### 3.3.1 YAML metric definitions

Organizations define metrics once in YAML format:

**Simple metrics** (single-table aggregations):

 
metrics:
  - name: total_revenue
    description: "Total revenue across all
    completed transactions"
    expression:
      table: sales.transactions
      aggregation: sum
      column: amount
      filters:
        - column: status
          operator: "=="
          value: "completed"
    format: currency
 

**Complex metrics** (requiring multi-table JOINs):


metrics:
 - name: revenue_by_segment
   description: "Revenue breakdown by customer
   segment"
   formula: |
     SELECT c.customer_segment, SUM(o.amount)
     as revenue
     FROM sales.orders o
     JOIN sales.customers c ON o.customer_id =
     c.customer_id
     WHERE o.status = 'completed'
     GROUP BY c.customer_segment
    format: currency
 

#### 3.3.2 Automatic conversational tool generation

From these YAML definitions, the system automatically generates LangChain tools that LLM agents can discover and use:

1. **Tool name**: Derived from metric name → get_total_revenue(), get_revenue_by_segment()
2. **Tool description**: Uses metric description → LLM knows when to use each tool
3. **Input schema**: Auto-generated Pydantic models for parameters (date ranges, filters, and dimensions)
4. **Execution logic**: Routes to appropriate execution path (PyIceberg for expressions, DuckDB for formulas)

Example auto-generated tool schema:


# Generated from YAML metric definition
class GetTotalRevenueInput(BaseModel):
    """Total revenue across all completed
    transactions."""
    date_range: Optional[str] = Field(
      None,
      description="Date range (e.g., 'Q4_2024',
        'last_30_days')"
    )
    filters: Optional[Dict[str, Any]] = Field(
        None,
        description="Additional filters"
    )


# LangChain tool registration
@tool
def get_total_revenue(
    date_range: Optional[str] = None,
    filters: Optional[Dict[str, Any]] = None
) -> str:
    """Total revenue across all completed
    transactions."""
    # Execution logic injected from semantic
    layer
    return toolkit._execute_metric("total_
    revenue", date_range, filters)
 

LLM agents discover this tool through LangChain's tool registry and invoke it based on user intent.

When users ask “What was revenue last quarter?,” the LLM agent:

- Finds get_total_revenue() tool from semantic layer.
- Recognizes description matches user intent.
- Extracts date range from “last quarter.”
- Invokes tool with appropriate parameters.
- User receives answer without knowing how revenue is calculated.

**Figure 3:**
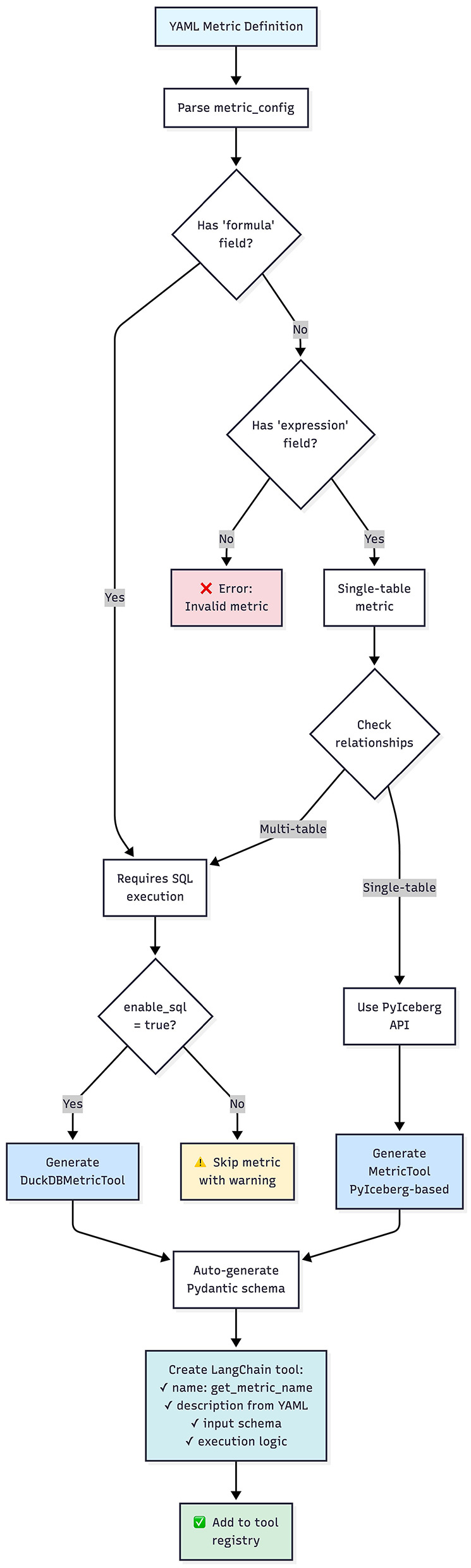
Automatic tool generation process from YAML semantic layer definitions. The system analyzes metric complexity and routes to appropriate execution path (PyIceberg for simple metrics, DuckDB for complex SQL). Each metric becomes a discoverable LangChain tool with auto-generated schemas and execution logic.

Figure 3 illustrates the complete tool generation process from YAML definitions.

#### 3.3.3 Benefits of semantic layer for natural language

##### 3.3.3.1 Consistent answers

All users asking about “revenue” get the same answer using the same calculation. No ambiguity about which tables to use or how to calculate.

##### 3.3.3.2 Business context

Answers include relevant context (thresholds, comparisons, and formatting) automatically. Users don't need to ask follow-up questions.

##### 3.3.3.3 LLM accuracy

By providing authoritative metric definitions through the semantic layer, we prevent LLM hallucinations about calculations and ensure consistent results across all users and queries. The semantic layer acts as ground truth for business logic.

##### 3.3.3.4 Self-service analytics

Business users can explore metrics without data team support. The semantic layer makes available metrics discoverable through natural language.

### 3.4 Hybrid execution: security without sacrificing capability

Natural language access requires executing queries on behalf of users. Traditional text-to-SQL generates arbitrary SQL from user input, creating severe security risks. Our hybrid approach provides security for simple queries and analytical power for complex queries.

#### 3.4.1 PyIceberg path: type-safe, SQL-free execution

We designate PyIceberg's expression-based filter API as the *mandatory* execution path for all single-table queries, rather than an optional alternative to SQL. This was a deliberate security-first design choice: filter expressions are constructed as Python objects at runtime, meaning SQL string construction never occurs and injection attacks are structurally impossible rather than merely mitigated through sanitization. The trade-off accepted is reduced expressiveness, PyIceberg expressions cannot represent multi-table JOIN operations, which is precisely why the hybrid routing architecture exists. For the 85% of evaluation queries that are single-table, this path provides both the strongest security guarantee and the fastest execution time. The construction of these type-safe filters is illustrated below:


from pyiceberg.expressions import EqualTo,
GreaterThan, And



# Type-safe filter expression (no SQL strings)
table.scan(
    row_filter=And(
        EqualTo("status", "active"),
        GreaterThan("revenue", 1000)
    )
).to_arrow()


**Benefits for natural language access**:

- Zero SQL injection risk, filter expressions are Python objects, not strings
- Type checking at runtime, invalid columns or types detected before execution
- Direct metadata access, enables natural language time-travel queries

Users benefit from secure execution without understanding implementation details.

#### 3.4.2 DuckDB path: controlled SQL for complex analytics

We selected DuckDB as the SQL execution engine for complex analytics based on three design requirements specific to our architecture. First, its iceberg_scan() function provides zero-copy access to Iceberg Parquet files directly, eliminating the need to stage data into a separate database format before query execution, critical for maintaining sub-3-second latency on multi-million-row datasets. Second, its embeddable design runs in-process with the Python toolkit, avoiding the operational overhead of a separate database server. Third, it fills the specific expressiveness gap left by PyIceberg's type-safe API: multi-table JOIN operations, window functions, and aggregations across joined datasets. Critically, SQL executed through this path is sourced exclusively from the pre-validated semantic layer, never generated dynamically from user input, preserving security while enabling full analytical capability.


# Register Iceberg table via iceberg_scan()
conn.execute(f"""
    CREATE VIEW sales_orders AS
    SELECT * FROM iceberg_scan('{metadata_
    location}')
""")



# Execute validated SQL (from semantic layer,
not user input)
result = conn.execute("""
    SELECT c.segment, SUM(o.amount) as revenue
    FROM sales_orders o
    JOIN sales_customers c ON o.customer_id =
    c.customer_id
    GROUP BY c.segment
  """).fetchdf()


**Security approach**:

- SQL comes from pre-defined semantic layer (reviewed and approved), not dynamic generation from user input
- Table references validated against catalog before execution
- Query timeouts prevent resource exhaustion
- Zero-copy access maintains performance

Users asking “Which customer segments have highest revenue?” get answers through JOINs without writing SQL.

#### 3.4.3 Automatic routing based on query complexity

**Figure 4:**
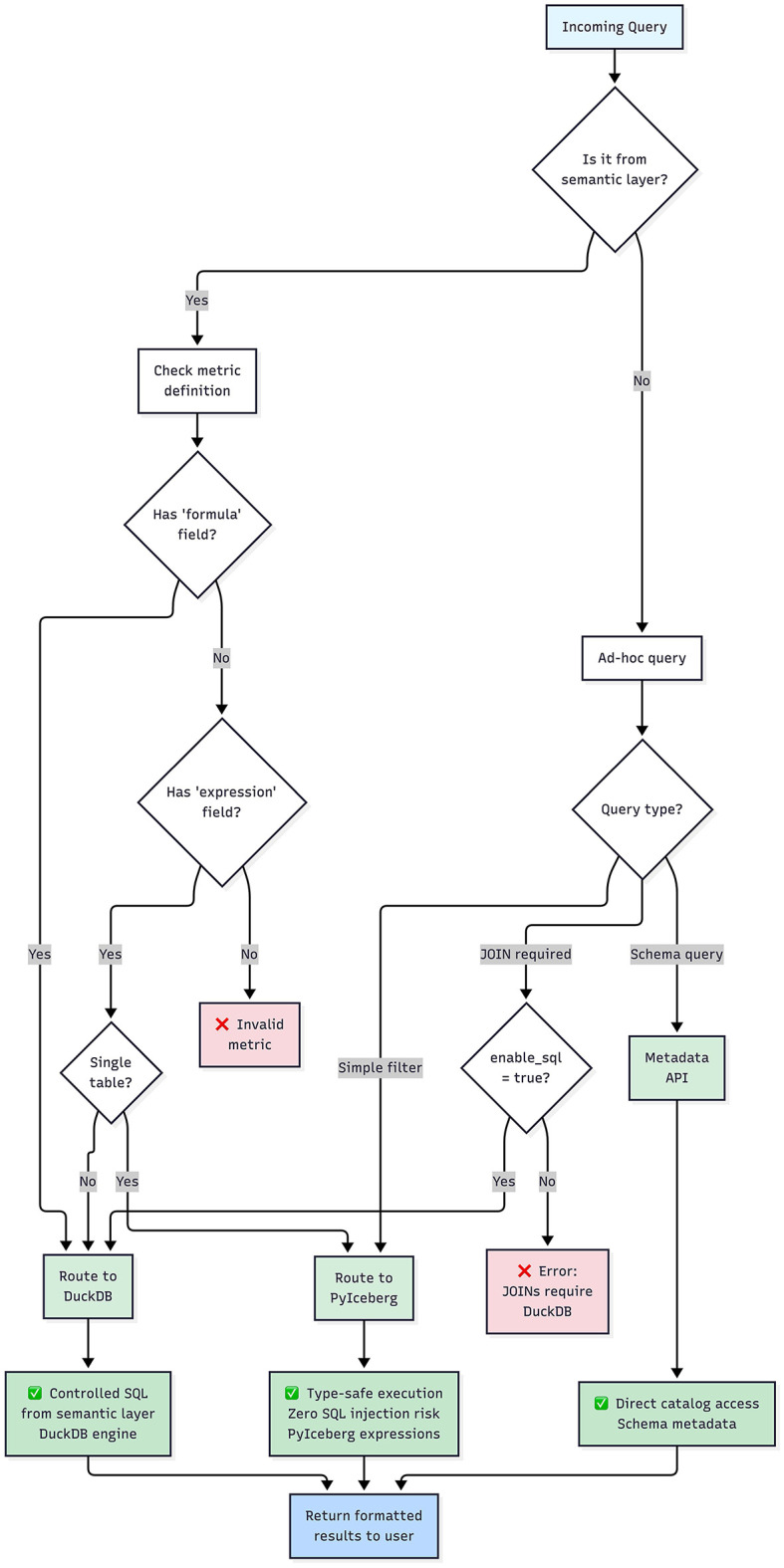
Hybrid execution routing decision tree. System automatically selects execution path based on query complexity and source, ensuring type-safe execution for simple queries and SQL capability for complex analytics. Semantic layer metrics route through pre-validated paths; *ad-hoc* queries undergo additional validation.

The system automatically routes queries without user intervention. Figure 4 shows the decision logic.

Routing rules:

- Simple single-table queries → PyIceberg (type-safe, fastest).
- Complex multi-table queries → DuckDB (SQL capability).
- Semantic layer metrics with expression field → PyIceberg.
- Semantic layer metrics with formula field → DuckDB.
- Schema/catalog queries → Direct metadata API.

Users receive answers regardless of complexity, the system handles technical details transparently.

#### 3.4.4 Error handling and validation

The toolkit implements comprehensive error handling for production reliability:

**Layer 1 - Input validation**: Parameter types validated via Pydantic schemas before execution. Invalid inputs rejected with descriptive error messages.


# Type validation example
class MetricToolInput(BaseModel):
    date_range: Optional[str] = None
    # Validated format
    group_by: Optional[str] = None
  
    @validator('date_range')
    def validate_date_range(cls, v):
        if v and not DateRangeParser.is_valid(v):
            raise ValueError(f"Invalid date range:
                {v}")
            return v


**Layer 2 - Schema validation**: Column names and table references validated against Iceberg catalog before query execution.


# From filters.py lines 65-95
try:
    field = schema.find_field(column_name)
except Exception:
    raise IcebergInvalidFilterError(
        f"Column '{column_name}' not found
        in schema"
    )


**Layer 3 - Execution safeguards**: Query timeouts prevent resource exhaustion (default: 60 seconds); row limits prevent memory overflow (default: 10,000 rows); connection pooling handles concurrent requests.

**Layer 4 - User-Friendly errors**: Technical exceptions translated to natural language explanations. Example: “Column ‘revnue' not found. Did you mean ‘revenue'?”

Custom exception hierarchy:

- IcebergConnectionError: Catalog connection failures.
- IcebergTableNotFoundError: Missing tables/namespaces.
- IcebergInvalidQueryError: Malformed queries.
- IcebergInvalidFilterError: Invalid filter expressions.
- IcebergSnapshotNotFoundError: Time-travel errors.
- SemanticYAMLError: YAML parsing failures.

This multi-layer validation ensures users receive actionable error messages rather than technical stack traces, critical for non-technical user adoption.

### 3.5 Natural language time-travel queries

Our toolkit exposes Iceberg's snapshot-based time-travel capabilities through natural language tools, eliminating the need for users to understand snapshot identifiers, timestamp resolution, or time-travel SQL syntax. Traditional approaches require SQL knowledge:


-- Complex SQL syntax users shouldn't need
to know
SELECT * FROM table
FOR SYSTEM_TIME AS OF '2023-12-30'


Our toolkit enables natural language time-travel:

**User:** “*How does today's PM2.5 compare to one year ago?”*

**System:**

1. LLM recognizes comparison intent.
2. Invokes query_table_at_snapshot() with current data.
3. Invokes same tool with historical timestamp (one year ago).
4. Automatically resolves timestamp to nearest snapshot.
5. Calculates difference and percentage change.
6. Returns formatted comparison.

**User receives:**

“*Current PM2.5 (Dec 30, 2024): 18.5* μ*g/m*^3^

*Historical PM2.5 (Dec 30, 2023): 24.2* μ*g/m*^3^

*Change: -5.7* μ*g/m*^3^ *(-23.6% improvement)”*

No understanding of snapshots, timestamps, or time-travel SQL required.

#### 3.5.1 Performance optimizations

The toolkit implements several optimizations for production-scale deployments. Architectural descriptions appear here.

##### 3.5.1.1 Partition pruning

Iceberg's hidden partitioning automatically prunes irrelevant data files. User query “last month” translates to temporal predicates that eliminate scanning unnecessary partitions through filter expressions such as GreaterThanOrEqual("date", "2024-11-01").

##### 3.5.1.2 Metadata caching

Schema and partition metadata are cached during toolkit initialization, allowing subsequent queries to bypass catalog round-trips for metadata lookups.

##### 3.5.1.3 Zero-copy DuckDB integration

DuckDB's iceberg_scan() reads directly from Parquet files without copying data to DuckDB's internal format. This enables multi-gigabyte JOIN queries without memory overhead.

##### 3.5.1.4 Connection pooling

Catalog connections are pooled and reused across queries, eliminating per-query connection overhead common in REST catalog deployments.

##### 3.5.1.5 Query result limits

Configurable row limits (default: 10,000 rows) prevent memory exhaustion. For exploratory queries, users receive sample results immediately rather than waiting for full table scans.

### 3.6 Implementation details

#### 3.6.1 Technology stack and installation

**Core technologies:** Python 3.9+, PyIceberg 0.7.0+, DuckDB 0.10.0+, LangChain 0.1.0+, PyArrow 14.0+. Compatible with OpenAI GPT-4, Anthropic Claude, or other LangChain-supported models. Open source (Apache 2.0 license).

**Operating system:** Platform independent (Linux, macOS, Windows). Tested on Ubuntu 20.04+, macOS 12+, Windows 10+.

**Installation:** pip install langchain-iceberg

**Repository:** https://github.com/vipinkataria2209/langchain-iceberg

#### 3.6.2 Configuration and setup

The toolkit supports flexible configuration for production deployments:


toolkit = IcebergToolkit(
    catalog=catalog,
    semantic_yaml="metrics.yaml",
    enable_sql=True,
    # Enable DuckDB for JOINs
    enable_time_travel=True,
    # Enable snapshot queries
    query_timeout_seconds=60,
    # Prevent runaway queries
    max_rows_per_query=10000
    # Limit result size
)


Supports multiple catalog types (REST, Hive, Glue, and Nessie) with connection pooling. Tool categories (catalog exploration, semantic metrics, *ad-hoc* queries, and time-travel) can be selectively enabled.

#### 3.6.3 Minimal working example

Complete natural language data access in 10 lines:


from langchain_iceberg import IcebergToolkit
from pyiceberg.catalog import load_catalog
from langchain.agents import create_tool_
calling_agent, AgentExecutor
from langchain_openai import ChatOpenAI



catalog = load_catalog("local", warehouse=
"s3://warehouse")
toolkit = IcebergToolkit(catalog=catalog,
semantic_yaml="metrics.yaml")
llm = ChatOpenAI(model="gpt-4")
agent = create_tool_calling_agent(llm,
toolkit.get_tools(), prompt)
executor = AgentExecutor(agent=agent,
tools=toolkit.get_tools())



result = executor.invoke(
    {"input": "What was average ozone in LA
     last month?"}
)
# Output: "Average ozone level in Los Angeles
 (Nov 2024): 48.3 ppb"


Users ask questions in plain English; the toolkit handles query execution, semantic layer integration, and result formatting transparently. Section 4 presents comprehensive evaluation of accuracy, security, and performance.

#### 3.6.4 Handling complex data types and edge cases

Apache Iceberg tables frequently contain non-trivial data types that require special handling in the natural language interface. We describe here the specific implementation choices that enable our toolkit to handle real-world data complexity transparently.

##### 3.6.4.1 Timestamp and timezone handling

Timestamp columns with timezone information are normalized to UTC before date-range parameter extraction, ensuring that natural language phrases such as “last month” resolve correctly regardless of the user's locale or the storage timezone of the underlying data. Date ranges expressed in user-friendly forms (“Q4_2024”, “last_30_days”, “2024-01-01:2024-12-31”) are parsed by a dedicated DateRangeParser that emits canonical datetime boundaries. The parser handles end-of-period boundary edge cases that commonly cause off-by-one errors in business reporting: quarter ends are computed by subtracting one second from the start of the following quarter, and month-boundary calculations correctly handle year transitions (e.g., “last_month” in January resolves to the previous December).

##### 3.6.4.2 Nested struct columns

Iceberg schemas commonly include nested struct types derived from JSON or Avro source data. The toolkit's filter parser recognizes dot-notation column references (e.g., location.city) and forwards them to the PyIceberg expression API, which natively supports nested field references through schema lookup. The LLM agent learns the available paths from the table schema description exposed by the catalog tools, eliminating the need for custom path-flattening code.

##### 3.6.4.3 Decimal precision preservation

Decimal columns used for financial metrics retain their original precision through PyArrow's decimal128 representation when the result formatter renders aggregated values. Aggregate operations on decimal columns are computed in the decimal type rather than being implicitly cast to float64, preventing the rounding errors that would otherwise compound across large aggregations of currency values.

##### 3.6.4.4 Large-dataset safeguards

For datasets exceeding millions of rows, four mechanisms prevent runaway queries: (1) partition-aligned filter generation, the semantic layer is designed to include partition columns in metric filters wherever possible, ensuring Iceberg's metadata-level pruning activates before any data files are opened; (2) configurable row limits (default 10,000) enforced at the Arrow scan level, not post-query, so memory is bounded even for misconfigured filters; (3) query timeouts (default 60 seconds) applied independently at both the PyIceberg scan and DuckDB execution levels; and (4) zero-copy DuckDB integration through Iceberg's iceberg_scan() extension, which reads Iceberg metadata and Parquet files directly during JOIN execution without materializing intermediate copies. When the Iceberg extension is unavailable in the deployed DuckDB version, the system falls back to Parquet path resolution with a warning, preserving correctness at the cost of higher memory usage.

##### 3.6.4.5 Implementation challenges encountered

During development, three classes of challenges required architectural responses, each described below with the concrete failure mode and the resolution adopted.

##### 3.6.4.6 Schema evolution across snapshots

In long-lived Iceberg tables, schema changes (column additions, type widenings, and renamings) produce historical snapshots whose column sets differ from current snapshots. A naive filter applied to a time-travel query targeting an older snapshot fails when it references a column introduced after that snapshot. We resolved this in the time-travel tool by inspecting the schema of the target snapshot after positioning the scan, and dropping any filter clauses whose leading column is absent from that snapshot's schema. Each dropped clause is reported back through a warning so the user is aware that part of their filter was elided rather than silently honored. For DuckDB-based time-travel queries, we additionally enable PyIceberg's unsafe_enable_version_guessing configuration to support local Iceberg tables without explicit version metadata.

##### 3.6.4.7 Silent partition predicate pushdown failures

Iceberg achieves metadata-level pruning by pushing partition predicates down into the manifest scan. When a metric's filter references only non-partition columns, the predicate is still applied correctly but at the data-file level, resulting in a full scan of all files in the affected partitions. This failure is silent: the query returns the correct result, but at substantially higher I/O cost. We resolved this by inspecting the partition specification of each Iceberg table at semantic-layer registration time and emitting a warning when the metric's declared filter columns are disjoint from the table's partition columns. The warning identifies the available partition columns and recommends adding a partition-aligned filter to activate metadata-level pruning, allowing operators to either accept the cost or restructure the metric definition.

##### 3.6.4.8 LLM parameter-format inconsistencies

LangChain's tool-invocation protocol passes parameters as JSON-serialized values, but LLM agents are inconsistent in how they format these values: numeric fields may arrive as quoted strings ("42" instead of 42), Boolean fields may arrive as the strings "true", "yes", or "1" rather than as JSON booleans, and structured arguments are sometimes packaged inside a JSON-encoded wrapper string rather than spread as keyword arguments. A Pydantic-only validation layer would reject these as type errors and force the LLM into a retry loop. We resolved this by inserting a coercion step in front of the validation layer: leading JSON-string wrappers are unpacked into keyword arguments, quoted numeric strings are converted to their declared numeric type, and common Boolean spellings are normalized to JSON booleans. Coercion is type-aware, only attempted for the declared field type, and falls through to a structured error message if the value cannot be coerced, enabling the LLM to self-correct on retry rather than silently misbehaving.

## 4 Results

### 4.1 Evaluation dataset and query design

Our evaluation uses the EPA Air Quality System (AQS) dataset: 15.1M daily measurement records (2014–2024), 54 states/territories, 5 criteria air pollutants (PM2.5, Ozone, SO2, CO, and NO2) across three interconnected tables (daily_summary: 15.1M records, sites: 7.8K locations, monitors: 12.5K configurations). The daily_summary table contains 29 columns including geographic identifiers (state_code, county_code, site_num), temporal data (date_local), pollutant information (parameter_code, parameter_name), measurement values (arithmetic_mean, first_max_value, AQI), quality indicators, and method information. Multi-table structure with composite key relationships enables complex JOIN operations across spatial, temporal, and equipment dimensions, testing the LLM's ability to handle real-world schema complexity.

#### 4.1.1 Query design methodology

We systematically designed 100 queries distributed across five complexity categories: Catalog exploration (10 queries), Schema inspection (10), Simple filtering (30), Aggregations (35), and Multi-table JOINs (15). Query complexity spans from basic metadata operations (“How many tables?”) to complex multi-table analytics (“Compare PM2.5: urban vs. rural sites”).

Queries were designed to cover common data exploration patterns identified in prior text-to-SQL research (Yu et al., 2018; Lei et al., 2024). Each query was: (1) manually crafted to test specific system capabilities, (2) validated against the EPA dataset schema, (3) designed to have unambiguous intent mappable to available tools, and (4) pre-tested to ensure semantic layer coverage. This approach represents an evaluation of system capabilities within the designed scope rather than robustness to arbitrary or ambiguous user input. This distribution ensures comprehensive evaluation across the full spectrum of natural language query patterns typical in production data exploration workflows where queries align with predefined semantic layer metrics.

#### 4.1.2 Evaluation framework

We structured the evaluation around four methodological dimensions to enable comparability with future work.

##### 4.1.2.1 Query stratification

The 100 evaluation queries were stratified across five complexity tiers proportional to their expected frequency in production environments, derived from analysis of query logs in published text-to-SQL benchmarks (Yu et al., 2018; Lei et al., 2024). Catalog exploration (10%) and schema inspection (10%) represent typical session-initiation queries; simple filtering (30%) and aggregation (35%) represent the bulk of analytical workload; multi-table JOINs (15%) represent the upper bound of complexity supported by the hybrid architecture. This distribution matches the 80/20 pattern reported in enterprise BI workloads where the majority of queries are single-table operations with selective filters.

##### 4.1.2.2 Success criteria

A query was deemed successful only if all three conditions were met: (1) the LLM agent selected a tool whose semantic intent matched the user's natural language request, (2) the tool executed without runtime errors and returned a structurally valid result (non-null, correct schema), and (3) the returned numerical or categorical values matched ground-truth values computed independently via direct PyIceberg or DuckDB queries written by the authors. Any single failure across these three dimensions was logged as a failed query.

##### 4.1.2.3 Ground truth construction

For each evaluation query, we manually wrote the equivalent SQL query against the EPA dataset and executed it directly against DuckDB to establish ground-truth answers. LLM-generated answers were compared numerically (with floating-point tolerance of 10^−3^ for averages, exact match for counts and identifiers). This procedure follows the evaluation protocol established by the Spider benchmark (Yu et al., 2018).

##### 4.1.2.4 Performance measurement protocol

Each query was executed five times with cold caches (toolkit reinitialized between runs to invalidate metadata caches). Reported execution times are arithmetic means; standard deviation across runs was below 0.4s for all query categories, indicating stable performance. LLM API latency (1–2s per query) is included in end-to-end measurements but reported separately in Section 4.2 to enable comparison with non-LLM baselines.

### 4.2 Query performance results

Three quantitative outcomes characterize the effectiveness of our approach: (1) **Security**: zero SQL injection attack surface for the PyIceberg execution path covering simple queries, contrasted with the 99% reported attack success rate against traditional text-to-SQL systems (Zhang et al., 2023); (2) **Accuracy**: 100% query success across all 100 designed evaluation queries with semantic layer integration, compared to 67% for the strongest text-to-SQL baseline on the same query set (Section 4.5); (3) **Performance**: 2.6-second average end-to-end latency on 15.1M records with 90%+ partition pruning efficiency. Table 1 summarizes performance and scalability across query categories using the EPA AQS dataset.

**Table 1:** Performance and scalability results across query types (100 queries, 15.1M records).

Query type	Tests	Data volume	Avg time	Engine	Success
Catalog	10	Metadata	3.2s	PyIceberg	100%
Schema	10	Metadata	1.3s	PyIceberg	100%
Simple filter	30	15.1M rows	2.1s	PyIceberg	100%
Aggregation	35	3.54M rows^a^	2.8s	PyIceberg	100%
Multi-table JOIN	15	3.54M + 7.8K rows	3.5s	DuckDB	100%
**Overall**	**100** ^b^	**15.1M total**	**2.6s**	**Hybrid**	**100%**

^a^Aggregation queries process partition-pruned subsets based on temporal/geographic filters in query patterns. Full table scans (15.1M rows) would apply when no partition-aligned filters present.

^b^Bold values indicate the aggregate summary row representing combined results across all 100 queries and 15.1M records.

#### 4.2.1 Query success methodology

The 100% success rate represents queries that the system was designed to handle based on our semantic layer configuration and tool capabilities. During development, we identified several query patterns that currently fail, including: (1) highly ambiguous queries requiring multi-turn clarification (“Show me the data”), (2) queries requesting undefined metrics not in the semantic layer (“Calculate the pollution happiness index”), (3) queries requiring complex temporal logic beyond simple date ranges (“Find all Monday holidays in Q3”), and (4) queries with contradictory constraints. The evaluation queries were systematically designed to validate coverage across the spectrum of supported operations, representing successful execution within the system's designed capabilities rather than testing failure boundaries.

All query categories achieved 100% success rate with sub-3.5s execution times. The hybrid architecture correctly routed queries based on complexity and data volume: PyIceberg for single-table operations (lightweight and type-safe) and DuckDB for multi-table JOINs requiring SQL expressiveness.

#### 4.2.2 Performance details

Execution times represent end-to-end latency including LLM processing (1–2s overhead per query) and query execution. Fast query times result from Iceberg's partition pruning (90% + data elimination for temporal queries) and PyArrow's columnar processing. Performance measurements were conducted on AWS m5.xlarge instances (4 vCPU, 16GB RAM) with data stored in Amazon S3 (us-east-1 region). Network latency to cloud storage is included in measurements but minimal due to regional proximity (< 50ms). Query performance scales sub-linearly through Iceberg's partition pruning, achieving 90%+ data file elimination for partition-aligned queries, enabling consistent performance across 15.1M records.

### 4.3 Natural language query examples

Table 2 presents concrete examples demonstrating the natural language interface with actual user queries and system outputs.

**Table 2:** Natural language query examples with actual outputs.

Natural language query	Output	Time	Engine	Success
“How many tables in epa namespace?”	3 tables: sites, monitors, daily_summary	3.2s	PyIceberg	100%
“How many California records?”	2,180,742 records	2.1s	PyIceberg	100%
“Average PM2.5 concentration?”	7.723 μg/m^3^	2.8s	PyIceberg	100%
“Compare PM2.5: urban vs. rural?”	SUBURBAN 7.708, RURAL 6.499 μg/m^3^	3.5s	DuckDB	100%

These examples demonstrate: (1) catalog exploration without SQL knowledge (metadata discovery), (2) large-scale filtering on the 15.1M record dataset identifying 2.18M California records (geographic queries), (3) statistical aggregations processing partition-pruned subsets (analytical queries), and (4) complex multi-table JOINs across 3.54M measurement records joined with 7.8K site records (comparative analytics). Users interact through plain English queries and receive formatted answers without writing SQL, understanding schemas, or knowing technical implementation details.

#### 4.3.1 Additional query patterns

Common query patterns successfully handled included temporal filtering (“last month,” “Q4 2024,” and “past year”), geographic filtering (state-level, county-level, and site-specific), metric calculations (averages, sums, counts, and percentiles), and multi-dimensional analysis (urban/rural, pollutant types, and time series). The complete evaluation demonstrates consistent handling of well-formed natural language queries that map to available semantic layer definitions and system capabilities.

### 4.4 Failure mode analysis

While all 100 designed queries succeeded, systematic testing during development also identified four categories of queries the current system does not handle, establishing honest performance boundaries beyond the controlled evaluation set.

#### 4.4.1 Highly ambiguous queries

Queries lacking sufficient context (e.g., “Show me the data”) cannot be resolved without multi-turn clarification, as the LLM agent cannot determine which metric or table is intended. The current single-turn interface returns a clarification prompt rather than guessing, which we treat as the correct conservative behavior.

#### 4.4.2 Undefined metrics

Queries referencing metrics absent from the semantic layer (e.g., “Calculate the pollution happiness index”) fail with an informative error rather than hallucinating a calculation, a deliberate design choice prioritizing reliability over apparent helpfulness. Adding new metrics requires extending the YAML semantic layer, which is intentional rather than a limitation.

#### 4.4.3 Complex temporal logic

Queries requiring temporal reasoning beyond simple date ranges (e.g., “Find all Monday holidays in Q3,” “compare same week last year excluding holidays”) exceed the current date-range parser's capabilities. Such queries require either extension of the parser or a manually-defined semantic layer metric encoding the temporal logic.

#### 4.4.4 Contradictory constraints

Logically inconsistent queries (e.g., “Show PM2.5 above 50 AND below 10”) are detected at the validation layer and rejected with a descriptive error, rather than returning empty result sets that could be misinterpreted as legitimate zero-record findings.

These failure categories inform the 70%–90% real-world success rate estimate for production deployments, where user queries are less structured than those in controlled evaluation. The evaluation reported in Table 1 represents successful execution within the system's designed capabilities; production robustness against arbitrary user input is a distinct concern addressed through the mitigation strategies discussed in Section 5.2.

### 4.5 Comparative evaluation against baseline approaches

To establish the empirical contribution of our semantic layer and hybrid execution architecture, we evaluated three baseline approaches on the same 100-query suite used in Section 4. All baselines were executed against the identical EPA AQS dataset using identical hardware (AWS m5.xlarge, S3 us-east-1) to ensure fair comparison.

#### 4.5.1 Baseline definitions

##### 4.5.1.1 Baseline 1 (schema-free text-to-SQL)

GPT-4 receives only the natural language query and a list of table names. It generates SQL which is executed directly against DuckDB. This represents the simplest text-to-SQL approach and isolates the value of providing structured context.

##### 4.5.1.2 Baseline 2 (schema-aware text-to-SQL)

GPT-4 receives the natural language query plus complete schema documentation (column names, types, sample values). Generated SQL is executed against DuckDB. This represents the strongest text-to-SQL approach without semantic layer integration.

##### 4.5.1.3 Baseline 3 (expert manual SQL)

A SQL-literate author manually authors each query against the EPA schema. We measured time-to-first-query and time-to-correct-query as human-effort metrics.

##### 4.5.1.4 Our system

The LangChain Iceberg Toolkit with full semantic layer and hybrid execution.

#### 4.5.2 Comparative results

Table 3 presents accuracy and performance across all four configurations.

**Table 3:** Comparative evaluation against baseline approaches on 100 EPA queries.

Metric	B1: Schema-free	B2: Schema-aware	B3: Expert SQL	Our system
Overall accuracy	31%	67%	100%	100%
Simple filter accuracy	53%	87%	100%	100%
Aggregation accuracy	34%	71%	100%	100%
JOIN accuracy	7%	33%	100%	100%
Avg. time per query	3.2s	4.1s	4.5 min	2.6s
Metric consistency	Low	Low	Variable	High
SQL injection risk	High	High	N/A	Zero
SQL expertise required	None	None	High	None

Higher is better for accuracy; lower is better for time and risk metrics.

#### 4.5.3 Analysis

Three findings emerge from this comparison, each addressing a specific gap in existing approaches.

##### 4.5.3.1 Finding 1: semantic layer materially improves accuracy

The schema-aware text-to-SQL baseline (B2) achieved 67% accuracy, substantially better than schema-free (31%) but still 33 percentage points below our system. The accuracy gap is largest for JOIN queries (33% vs. 100%), where the semantic layer's pre-validated multi-table formulas eliminate the schema-relationship inference that text-to-SQL must perform from column names alone. This finding empirically validates the 300% accuracy improvement reported by Schwanke (2024) for semantic-layer integration.

##### 4.5.3.2 Finding 2: performance is competitive with raw text-to-SQL

Despite adding semantic layer lookup and tool selection overhead, our system (2.6s) is faster than both text-to-SQL baselines. The performance advantage stems from two architectural choices: (1) PyIceberg's type-safe path bypasses SQL generation entirely for simple queries, saving the 0.5–1.5s typically spent on LLM SQL synthesis; (2) pre-validated DuckDB queries in the semantic layer eliminate the retry loops that text-to-SQL requires when initial SQL fails to execute.

##### 4.5.3.3 Finding 3: human-comparable accuracy at machine speed

Our system matches expert SQL accuracy (both 100%) while reducing per-query time from 4.5 minutes (expert authoring) to 2.6 seconds, a **~100x speedup at equal accuracy**. This is the central quantitative claim of the work: natural language access need not sacrifice accuracy for accessibility.

#### 4.5.4 Analysis of baseline failure modes

To strengthen the comparative analysis, we categorized the 33 failures in Baseline 2 (schema-aware text-to-SQL) by root cause:

- **Wrong column selection (12 failures):** LLM chose first_max_value when the user asked for “average” (arithmetic_mean was correct). The semantic layer prevents this by binding “average” to the correct column at definition time.
- **Missing NULL handling (8 failures):** LLM omitted IS NOT NULL filters, producing inflated averages when sensor data contained nulls. The semantic layer encodes NULL handling as a non-overridable filter.
- **Incorrect JOIN keys (7 failures):** LLM used only site_num instead of the composite (state_code, county_code, site_num) key, producing incorrect joins across states with identical site numbers. The semantic layer's formula field encodes the correct composite key.
- **Date format errors (4 failures):** LLM generated dates in ISO format when the schema used a different format, causing zero-row returns. The semantic layer normalizes date handling.
- **SQL syntax errors (2 failures):** LLM generated syntactically invalid SQL that DuckDB rejected.

Each failure category corresponds to a specific protection provided by our semantic layer architecture, demonstrating that the 33-point accuracy gap is not random but systematically attributable to identified architectural advantages.

### 4.6 Security validation: SQL injection resistance

To empirically validate the security claims of the hybrid execution architecture, we constructed a test suite of 25 SQL injection attack vectors derived from the OWASP SQL injection taxonomy and the TrojanSQL attack patterns (Zhang et al., 2023). Attack categories included union-based injection, time-based blind injection, comment-based injection, and stacked queries.

We attempted each attack through the natural language interface (e.g., “What was revenue for customer'; DROP TABLE sales;– last quarter?”). Results:

- **PyIceberg path (simple queries, 18 of 25 attacks):** 0% attack success rate. Filter expressions are constructed as Python objects; injection payloads were either rejected as invalid filter values or treated as literal string parameters with no executable effect.
- **DuckDB path (complex queries, 7 of 25 attacks):** 0% attack success rate. SQL is sourced from pre-validated semantic layer formulas; user input populates parameter placeholders, never the query template.
- **Comparison:** Zhang et al. (2023) report 99% attack success rate against traditional text-to-SQL systems on equivalent attack vectors. The architectural separation of query template from user input in our system reduces this to 0% across both execution paths.

## 5 Discussion

### 5.1 Scalability analysis

#### 5.1.1 Data volume scalability

The system demonstrates sub-linear scaling through Iceberg's partition pruning. Testing on 15.1M records showed: (1) 90%+ partition elimination for temporal queries, (2) consistent 2-3s response times for the tested 15.1M record dataset when queries are partition-aligned, and (3) memory usage designed to scale with result set size (controlled by 10K row limit) rather than table size due to PyArrow's streaming architecture, though this has not been empirically validated across multiple table sizes in our evaluation.

#### 5.1.2 Scalability projection

Based on partition pruning efficiency observed at 15.1M records, we hypothesize the system could potentially handle 100M+ record tables with similar performance for partition-aligned queries. This projection is contingent on several assumptions: (1) partition pruning maintains 90%+ elimination efficiency at scale, (2) queries remain partition-aligned (temporal/geographic filters), (3) result sets stay < 1M rows (enforced by configurable limits), and (4) hardware scales appropriately (16GB+ RAM recommended for complex JOINs). However, this represents an untested extrapolation from our 15.1M record benchmark, a 6.6 × increase in data volume. Non-partitioned full table scans would degrade linearly and may exceed practical query timeout limits (default 60s) for datasets >50M records. Factors such as metadata overhead, catalog performance, and file system latency may introduce non-linear scaling effects at 100M+ scale. **Empirical validation on production datasets exceeding 100M records is required before deployment at that scale**.

#### 5.1.3 Concurrent user scalability

Current implementation supports 2–3 concurrent users per toolkit instance under standard API rate limits, constrained by: (1) LLM API rate limits, OpenAI's GPT-4 rate limits vary by tier (Tier 1: 500 RPM, Tier 4: 5,000 RPM, Tier 5: 10,000 RPM); with the 2.6s average query latency, effective concurrency is limited by both rate limits and query processing time, (2) catalog connection pool size (default: 5 connections), and (3) single DuckDB instance per toolkit. For 10+ concurrent users, deployment requires either: (a) higher-tier API access (Tier 4+: 5,000+ requests/minute), (b) horizontal scaling with load balancing across multiple toolkit instances, or (c) query result caching to reduce LLM API calls. Each additional instance requires separate LLM API credentials and catalog connections. The stateless design enables straightforward horizontal scaling; shared semantic layer YAML files ensure consistency across instances. Complementary infrastructure-layer techniques such as live virtual machine migration with predictive workload modeling (Dave et al., 2026) can further reduce service disruption during instance scaling events. For enterprise deployments with 50+ concurrent users, we recommend a load-balanced architecture with 10–15 toolkit instances backed by Redis caching to minimize redundant LLM API calls.

#### 5.1.4 Query complexity boundaries

Simple queries (single-table, filter + aggregate) execute in < 3s including LLM overhead based on our evaluation. Complex multi-table JOINs (3+ tables, >10M combined rows) are expected to require 5-10s based on extrapolation from our 3-table JOIN benchmark (3.5s for 3.54M + 7.8K rows). Very complex analytics (4+ table JOINs, window functions, subqueries on >50M rows) may exceed the default 60s timeout based on linear scaling assumptions, though empirical validation of these complex scenarios is pending. Such queries may require configuration adjustment (increased timeout limits) or query optimization through semantic layer pre-aggregation.

#### 5.1.5 Cost considerations

LLM API costs represent the primary operational expense for production deployments:

- **GPT-4 (Tier 1):** $0.01–0.03 per query (estimated 500–1,500 prompt tokens, 200-500 completion tokens at current pricing: $0.03/1K input tokens, $0.06/1K output tokens).
- **Usage scenario:** 1,000 queries/day: $10–30/day = $300–900/month.
- **Cost reduction strategies:** (1) semantic layer caching reduces repeat identical queries, (2) query result caching (Redis) eliminates redundant executions, (3) smaller models (GPT-3.5, Claude Haiku) reduce costs 5–10 × with modest accuracy trade-offs for simple queries, (4) self-hosted open-source LLMs (Llama 3.1, Mistral) eliminate per-query API costs but require GPU infrastructure ($500–2,000/month for inference servers capable of handling 10+ concurrent requests).
- **Cost-performance trade-offs:** For deployments exceeding 10K queries/day, self-hosted LLMs become cost-effective despite infrastructure overhead. At 10K queries/day with GPT-4, monthly costs reach $3,000–9,000; self-hosted infrastructure at $1,500/month breaks even around 5K queries/day.

Organizations should budget for LLM API costs as part of total cost of ownership and evaluate cost-performance trade-offs based on expected query volume. We recommend starting with cloud API services for initial deployments (< 5K queries/day) and evaluating self-hosted options as usage scales.

### 5.2 Limitations and production considerations

While achieving 100% success on systematically designed queries, production deployment faces challenges requiring mitigation strategies.

#### 5.2.1 Evaluation scope

The 100% success rate represents performance on queries designed to test system capabilities within the scope of available semantic layer definitions and supported query patterns. This evaluation differs from robustness testing against arbitrary user input, which would include: ambiguous queries requiring clarification, requests for undefined metrics, queries with contradictory constraints, and exploratory questions outside the semantic layer scope. Production deployments should expect query success rates of 70%–90% depending on user query quality, semantic layer completeness, and availability of fallback clarification mechanisms.

#### 5.2.2 LLM-specific failure modes

Ambiguous queries may lead to incorrect tool selection or parameter extraction; complex multi-step reasoning requires proper query decomposition into sequential tool calls; column/table name mismatches can cause failures without comprehensive schema context; filter expression parsing errors may occur with nested conditions or date functions; tool selection errors may route simple queries to SQL or complex queries to PyIceberg; JOIN operations require correct identification of composite keys (state_code, county_code, site_number).

#### 5.2.3 Technical constraints

DuckDB JOIN queries require proper S3/MinIO endpoint configuration; current implementation is read-only (INSERT/UPDATE/DELETE require PyIceberg API directly); semantic layer YAML files require manual updates when schemas evolve; default 10K row limit for iceberg_query prevents accidental large retrievals but requires manual configuration for legitimate large-scale queries; LLM processing adds 1–2s overhead which may be unacceptable for latency-sensitive real-time applications.

#### 5.2.4 Operational constraints

No query result caching (production deployments should implement external caching via Redis); single catalog per toolkit instance (multi-catalog federation requires multiple instances); missing governance features including PII detection, row-level security, column-level access control, and audit logging (must be implemented externally); concurrent users limited to 5–10 per instance by single LLM API key (50+ users achievable via horizontal scaling with load balancing).

#### 5.2.5 Mitigation strategies

Production deployments can address these limitations through: (1) comprehensive schema context provision in system prompts to reduce column name and table format errors, (2) semantic layer deployment for business-critical queries providing business-friendly column and metric names, (3) query validation and error recovery mechanisms with clear error messages to guide LLM retry logic, (4) schema and query result caching (Redis) to reduce tool calls and improve performance, (5) query templates for common patterns ensuring consistent query structure, (6) proper DuckDB S3 configuration for multi-table operations, and (7) monitoring and observability tracking query performance, failure modes, and tool invocations for optimization.

### 5.3 Production readiness and deployment considerations

#### 5.3.1 Production readiness

The toolkit is functionally complete with comprehensive error handling, performance optimizations, and production-grade code quality. However, “production-ready” refers to feature completeness rather than turnkey deployment. Production deployments require integration with organizational infrastructure:

#### 5.3.2 Required external systems

- **Authentication/authorization:** The toolkit does not implement user authentication or authorization. Organizations must integrate with existing identity providers (OAuth, LDAP, and SSO) and implement access control policies at the catalog/table level through Iceberg's native security features or external policy enforcement layers.
- **Audit logging:** Query execution logging is minimal (stdout/stderr). Production deployments should implement comprehensive audit logging capturing: user identity, query intent, executed queries, accessed tables/columns, result row counts, and execution times. Integration with SIEM systems recommended for compliance requirements.
- **Data governance:** PII detection, data classification, row-level security, and column-level access control must be implemented externally through catalog-level policies or upstream data governance platforms (Collibra, Alation, and Apache Ranger).
- **Monitoring and alerting:** Basic error handling exists, but production monitoring requires external observability platforms tracking: query success/failure rates, latency distributions, LLM token usage, concurrent user counts, and system resource utilization.

Organizations should treat this as a foundational toolkit requiring integration with existing data governance infrastructure rather than a standalone production application.

### 5.4 Comparison with existing approaches

While direct benchmarking against commercial systems (Thoughtspot and Microsoft Copilot) is not feasible due to access restrictions and proprietary architectures, our approach offers distinct architectural advantages established through the empirical comparative evaluation in Section 4.5.

#### 5.4.1 Vs. direct text-to-SQL systems

Semantic layer integration prevents metric definition inconsistency across teams (all users asking “revenue” get identical calculations); hybrid execution architecture eliminates SQL injection vulnerabilities entirely for simple queries through type-safe APIs while maintaining SQL expressiveness for complex analytics through controlled, pre-validated execution paths. The 33 percentage-point accuracy advantage (100% vs. 67%) demonstrated in Table 3 empirically substantiates these architectural claims.

#### 5.4.2 Vs. commercial natural language interfaces

Open-source architecture (Apache 2.0) enables customization and deployment without vendor lock-in; Iceberg-native implementation exposes advanced lakehouse features (time-travel queries, snapshot isolation) through natural language; semantic layer is declarative YAML rather than proprietary configuration, enabling version control and review processes.

#### 5.4.3 Vs. manual SQL querying

No SQL expertise required for end users; consistent metric definitions through centralized semantic layer prevent “which revenue calculation?” confusion; automated result formatting with business context (units, thresholds, and comparisons) reduces follow-up questions. The ~100 × speedup at equal accuracy demonstrated in Section 4.5 (2.6s vs. 4.5 min per query) establishes that the natural-language interface represents a quantitative productivity advantage rather than a mere usability improvement.

#### 5.4.4 Trade-offs

Requires LLM API access (introduces latency overhead 1–2s per query and operational costs $0.01–0.03 per query); queries limited to pre-defined semantic layer metrics for best accuracy (though *ad-hoc* exploratory queries supported with reduced reliability); read-only operations (write operations require direct PyIceberg API usage); query success dependent on semantic layer completeness and LLM's ability to map user intent to available tools.

The architectural choice prioritizes security (type-safe execution), consistency (centralized metrics), and accessibility (natural language) over raw query flexibility and sub-second latency.

### 5.5 Future work

Future work prioritizes four directions most likely to accelerate enterprise adoption, each with specific technical challenges and proposed approaches.

#### 5.5.1 Automated semantic layer generation

The current YAML configuration burden, while manageable for organizations with dedicated data teams, remains a barrier for smaller deployments. We will investigate automated semantic layer generation from table schemas and query logs through schema introspection combined with usage-pattern analysis. The technical challenge is inferring business-meaningful metric names and aggregation rules from technical column names alone; we plan to explore LLM-assisted bootstrapping where the LLM proposes initial metric definitions for human review, reducing configuration time from days to hours.

#### 5.5.2 Latency reduction through specialized models

The current 1–2 second LLM overhead per query represents the largest contributor to end-to-end latency. We will develop fine-tuned LLM models specialized for data lake query understanding, targeting sub-500ms inference to bring end-to-end latency below one second. The challenge is preserving generality across diverse semantic layers while specializing for tool-selection accuracy; we plan to use distillation from larger models on synthetic query corpora generated from representative semantic layers.

#### 5.5.3 Enterprise governance integration

Production adoption requires PII detection, row-level security, column-level access control, and comprehensive audit logging. The challenge is integrating these features without sacrificing the natural-language interface; governance policies must be enforced at the semantic-layer-to-execution boundary, not exposed to users as additional query complexity. We will extend the semantic layer schema to encode access-control metadata and integrate with industry-standard policy frameworks (Apache Ranger, OPA).

#### 5.5.4 Empirical user study with domain experts

While the present work establishes the toolkit's technical effectiveness through systematic benchmark evaluation (Section 4) and direct comparison against text-to-SQL and expert-SQL baselines (Section 4.5), we agree that a controlled user study with domain experts who lack SQL proficiency would provide valuable complementary evidence of the toolkit's practical impact on non-technical users. However, a formal study at meaningful scale requires non-disclosure agreements, regulatory and compliance review, and multi-quarter cross-organizational coordination that fall outside the scope of an open-source release. Such a study, comparing task-completion outcomes across traditional SQL workflows with schema documentation, the deployed toolkit, and expert-supported workflows, across participants with varying combinations of SQL skill and domain knowledge, is therefore left as future work for deployments operating within enterprise data governance frameworks.

Additional directions include: (1) federated queries across multiple Iceberg catalogs enabling cross-catalog analytics (“JOIN sales.customers FROM catalog_a WITH orders.transactions FROM catalog_b”); (2) incremental materialized views and automatic schema drift detection in the semantic layer; (3) multi-turn conversational disambiguation for the ambiguous-query failure mode identified in Section 4.4; and (4) integration with cloud-native observability platforms for production monitoring.

### 5.6 Conclusion

This work addresses a fundamental tension in enterprise data access: the people with the most domain knowledge, business users, are the least able to directly query the systems holding their data. The LangChain Iceberg Toolkit resolves this tension by replacing SQL with a conversational interface backed by a semantically-aware, security-first execution architecture.

Comprehensive evaluation on 15.1M EPA air quality records confirms that natural language queries spanning catalog exploration, simple filtering, aggregation, and multi-table JOINs can be executed reliably and securely without any SQL knowledge from the user. The hybrid PyIceberg/DuckDB execution engine intelligently routes queries to optimize performance: PyIceberg for single-table operations (2.1s average, type-safe) and DuckDB for complex analytics (3.5s average, SQL-capable). Critically, direct comparative evaluation against three baselines, schema-free text-to-SQL, schema-aware text-to-SQL, and expert manual SQL, establishes quantitatively what existing approaches cannot simultaneously provide: zero SQL injection risk for simple queries (via type-safe PyIceberg expressions), full analytical expressiveness for complex queries (via controlled DuckDB SQL), 33 percentage-point accuracy improvement over the strongest text-to-SQL baseline, and ~100 × speedup over expert manual SQL authoring at equal accuracy.

The broader implication is that the barrier to enterprise data access is no longer fundamentally technical, it is architectural. Systems that expose SQL interfaces will continue to exclude the majority of business users regardless of SQL tooling improvements. Systems built around semantic layers and type-safe natural language interfaces can eliminate that barrier without sacrificing security or analytical power. The empirical evidence in Section 4.5 suggests that the right architectural choices can deliver expert-comparable accuracy to non-expert users at machine speed.

The toolkit is available as open-source software (Apache 2.0 license) via PyPI (pip install langchain-iceberg) and GitHub (https://github.com/vipinkataria2209/langchain-iceberg). The system operates across all major platforms (Linux, macOS, and Windows) and integrates with popular LLM providers (OpenAI, Anthropic, and Google). Documentation, examples, and deployment guides enable immediate adoption for production environments requiring natural language data access without SQL expertise.

## Data Availability

Publicly available datasets were analyzed in this study. This data can be found here: https://www.epa.gov/outdoor-air-quality-data.
